# Skeletal muscle loss during neoadjuvant chemotherapy predicts poor prognosis in patients with breast cancer

**DOI:** 10.1186/s12885-022-09443-1

**Published:** 2022-03-26

**Authors:** Masatsugu Amitani, Takaaki Oba, Nami Kiyosawa, Hiroki Morikawa, Tatsunori Chino, Ai Soma, Tadafumi Shimizu, Koichi Ohno, Mayu Ono, Tokiko Ito, Toshiharu Kanai, Kazuma Maeno, Ken-ichi Ito

**Affiliations:** Division of Breast and Endocrine Surgery, Department of Surgery, Shinshu University School of Medicine, 3-1-1 Asahi, Matsumoto, Nagano, Japan

**Keywords:** Skeletal muscle index, Neoadjuvant chemotherapy, Breast cancer, Disease-free survival

## Abstract

**Background:**

The skeletal muscle index (SMI), which is calculated as the ratio of skeletal muscle area at the third lumbar vertebral level divided by height squared, has been considered a prognostic factor in patients with breast cancer. However, the prognostic impact of changes in SMI during treatment remains unclear. This study aimed to evaluate the influence of SMI changes in patients with breast cancer undergoing neoadjuvant chemotherapy (NAC).

**Methods:**

We reviewed patients with breast cancer who underwent NAC and subsequent surgery for breast cancer between 2010 and 2017. The rate of SMI change during NAC was calculated, and the association between SMI changes and prognosis was retrospectively analyzed.

**Results:**

In total, 141 patients were evaluated. 48 (34.0%), 53 (37.6%), and 40 (28.4%) patients exhibited increased (≥ 3%), maintained (− 3% <, < 3%), and decreased (− 3% ≥) SMI during NAC, respectively. The decreased SMI group showed significantly poorer disease-free survival than the maintained and increased SMI groups (hazard ratio [HR] 8.29, *p* <  0.001 for the decreased vs. increased SMI groups; HR 3.49, *p* <  0.001 for the decreased vs. maintained SMI groups). Moreover, decreased SMI was an independent risk factor for disease-free survival in multivariate analysis (HR 3.68, *p* <  0.01).

**Conclusions:**

Skeletal muscle loss during NAC predicts poor prognosis. Our results underscore the importance of monitoring and maintaining skeletal muscle mass during NAC.

**Supplementary Information:**

The online version contains supplementary material available at 10.1186/s12885-022-09443-1.

## Background

Neoadjuvant chemotherapy (NAC) has become a standard treatment option for patients with locally advanced but operable breast cancer [1]. NAC can downstage a primary tumor and axillary lymph nodes, which enables us to perform breast-conserving surgery in patients who might need a mastectomy at initial diagnosis. Furthermore, different from adjuvant chemotherapy, NAC also offers important clinical information, including response to chemotherapeutic agents and pathological complete response (pCR) rate, which has been demonstrated to be a prognostic marker in human epidermal growth factor receptor type 2 (HER2)-positive or triple-negative (TN) breast cancer [2]. In addition, NAC allows the evaluation of dynamic changes in the systemic conditions of patients who have a primary tumor during treatment*.* We previously demonstrated that a decrease in the prognostic nutritional index (PNI), which represents systemic nutritional and immunological status, during NAC had a prognostic impact in patients with breast cancer [3]. Hence, NAC may have the potential to provide other prognostic markers with respect to changes in patients’ status during treatment.

The influence of body weight and composition on patient outcomes has been a focus in the field of breast cancer studies. Previous studies have indicated that high body mass index (BMI) was associated with poor prognosis in patients with breast cancer [4, 5]. Conversely, some studies have shown no correlation between BMI and patient outcomes in breast cancer [3, 6]. This uncertainty underlying the influence of BMI is possibly, at least in part, because of the fact that BMI cannot account for the difference between adipose tissue and muscles and thus cannot evaluate the exact body composition. Certainly, the proportions of visceral fat, subcutaneous fat, and skeletal muscle vary significantly between individuals with the same BMI [7]. Thus, there is an unmet need for more accurate markers for estimating body composition, and skeletal mass volume was established as one of the markers for this [8].

Sarcopenia, defined as a condition with loss of muscle mass in older adults [9], is correlated with poor survival in various solid malignancies, including breast cancer [8, 10–16]. In addition to the importance of sarcopenia at the beginning of cancer treatment, skeletal muscle loss during palliative chemotherapy has recently been shown to be associated with reduced survival in patients with gastrointestinal cancer [17–22]. However, there is currently limited knowledge of changes in muscle mass during NAC and their impact on prognosis in patients with breast cancer. Generally, breast cancer is more likely to occur in younger patients than other types of cancer, such as lung, gastric, and colorectal cancers [23]. Furthermore, unlike patients undergoing palliative chemotherapy for other types of cancer, those treated with NAC for breast cancer have early-stage cancer. Hence, patients with breast cancer undergoing NAC are not likely to be sarcopenic at diagnosis [3]. Accordingly, it is possible that the effect of skeletal muscle index (SMI) changes in patients with breast cancer undergoing NAC is not equivalent to that in patients with other types of cancer undergoing palliative chemotherapy.

This study aimed to investigate the prognostic impact of changes in skeletal muscle mass during NAC in patients with breast cancer. To this end, we evaluated the changes in SMI during NAC in patients and investigated their association with patient outcomes.

## Methods

### Patients and study design

This retrospective study assessed patients with breast cancer with a performance status (Eastern Cooperative Oncology Group performance status [24]) of 0 who underwent NAC and subsequent surgery at Shinshu University Hospital between February 2010 and December 2017. The inclusion criteria were as follows: (1) patients who had pathologically confirmed breast cancer by core needle biopsy and (2) those who underwent computed tomography (CT) or ^18^F-fluorodeoxyglucose positron emission tomography/CT (^18^F-FDG-PET/CT) within both 4 weeks of initiation of NAC and surgery following NAC. Patients who could not complete NAC owing to chemotoxicity or disease progression during NAC (*n* = 14) were excluded. A total of 141 patients were included in this study. This study conformed to the provisions of the Declaration of Helsinki (64th WMA General Assembly, Fortaleza, Brazil, October 2013). The study was approved by the local ethics committee on the clinical investigation of Shinshu University (no. 5037). Because this was a retrospective study of anonymized data, the need for informed consent was waived.

### Data collection

Clinical information, including age, height, body weight, sex, menopausal status, clinical stage at diagnosis, histological type, histological grade (HG), estrogen receptor (ER) status, progesterone receptor (PgR) status, HER2 status, lymph node metastasis, NAC regimens, surgical procedure, pathological responses to NAC, and presence of recurrence, were collected from the patients’ medical records. ER, PgR, and HER2 statuses were examined in samples collected by pretreatment core needle biopsy. Subtypes of breast cancer were defined as follows: luminal (ER or PgR positive and HER2 negative), luminal HER2 (ER or PgR positive and HER2 positive), HER2-enriched (ER and PgR negative, HER2 positive), and TN (ER, PgR, and HER2 negative). HG was determined according to the Scarff–Bloom–Richardson grading system. Lymph node metastasis was determined by fine-needle aspiration cytology (FNA), but some patients were considered positive for lymph node metastasis without FNA if abnormal swelling of lymph nodes in CT findings or strong uptake of ^18^F-FDG on PET/CT findings was apparent. Disease-free survival (DFS) was defined as the time from surgery to the date of detection of locoregional relapse or distant metastases, whichever occurred first. Overall survival (OS) was assessed from the day of surgery to the date of death from any causes.

Skeletal muscle area (SMA) was measured using CT or ^18^F-FDG-PET/CT images before NAC and surgery. ^18^F-FDG PET/CT scans were taken at the Ichinose Neurosurgical Hospital (Matsumoto, Nagano, Japan) with a standard technique using a Discovery ST Elite Performance scanner (GE Healthcare Japan, Tokyo, Japan). Attenuation-corrected images were reconstructed in the coronal plane. SMA was measured as the cross-sectional area of the surrounding muscles (i.e., psoas, paraspinals, transversus abdominis, rectus abdominis, and internal and external obliques) by semi-automatic tracing using images at the third lumbar vertebral level (L3) visualized within a range of − 29 to 150 Hounsfield units using the EV Insite R (PSP Corporation, Tokyo, Japan) system and expressed in cm^2^. SMI was calculated as the ratio of SMA divided by height squared (m^2^), whereas BMI was calculated as the patient’s body weight (kg) divided by height squared (m^2^). The percent change in SMI and BMI was calculated as the percent change in each parameter after NAC from that at pretreatment. The serum albumin (Alb) (g/dl) and neutrophil-to-lymphocyte ratio (NLR), which was calculated as the total neutrophil count divided by the total lymphocyte counts, were also obtained from pre- and post-NAC blood examination. The change of Alb and NLR value during NAC were calculated as each value on post-NAC minus that on pre-NAC.

### NAC regimens and surgical methods

Two different NAC regimens were used: (1) triweekly administered anthracycline-based fluorouracil-epirubicin-cyclophosphamide (FEC) (500 mg/m^2^ fluorouracil, 100 mg/m^2^ epirubicin, and 500 mg/m^2^ cyclophosphamide) regimen, except for one patient who had been treated with an EC (60 mg/m^2^ epirubicin and 600 mg/m^2^ cyclophosphamide) regimen in another hospital and was transferred to our hospital for surgery, and (2) taxane regimens, including triweekly administered docetaxel (DTX) 75 mg/m^2^ or weekly administered paclitaxel (PTX) 80 mg/m^2^. Four cycles of DTX or PTX was administered following four cycles of EC/FEC. In HER2-positive patients, 6 mg/kg (triweekly) or 2 mg/kg (weekly) trastuzumab was administered simultaneously with a taxane regimen. For the EC/FEC regimen, dexamethasone (12 mg) and granisetron hydrochloride (3 mg) or palonosetron hydrochloride (0.75 mg) were infused intravenously before the administration of chemotherapeutic agents. Together with these drugs, aprepitant (125 mg) was orally administered to avoid chemotherapy-induced nausea and vomiting since 2011. Concomitantly, dexamethasone (8 mg) was administered orally one and 2 days after treatment. In addition, aprepitant (80 mg) or granisetron hydrochloride (2 mg) was orally administered from 1 day after treatment for two (aprepitant) or five (granisetron hydrochloride) consecutive days. For PTX and DTX treatment, 8 mg of dexamethasone was administered intravenously prior to treatment. In addition, for patients treated with DTX, 8 mg of dexamethasone was administered 1 day before treatment and 1 day and 2 days after treatment (a total of 3 days) orally. Surgery was performed within 4–7 weeks after NAC completion. All patients underwent axillary lymph node dissection. The efficacy of NAC was pathologically examined in surgical specimens, and pCR was defined as no evidence of residual invasive carcinoma in the breast tissue, regardless of the axillary lymph node status.

### Adjuvant trastuzumab, endocrine, and radiation therapy after surgery

Following surgery, extensional adjuvant trastuzumab (initially 8 mg/kg, followed by 6 mg/kg) was administered every 3 weeks for 12 months to patients with HER2-positive breast cancer. Whole breast irradiation of 50–60 Gy was performed for patients who underwent breast-conserving surgery, whereas chest wall and regional lymph node irradiation of 50–60 Gy was performed for patients with more than three nodal metastases on postoperative pathological examinations or preoperative imaging examinations, including ultrasonography, magnetic resonance imaging, or ^18^F-FDG-PET/CT. In addition, postmenopausal patients with positive ER or PgR status were treated with aromatase inhibitors for more than 5 years, whereas premenopausal patients were treated with tamoxifen or tamoxifen with luteinizing hormone-releasing hormone agonist.

### Statistical analyses

Categorical variables were analyzed using the chi-squared test, whereas continuous variables were analyzed using two-sided *t-*tests or one-way analysis of variance with Tukey’s multiple comparisons. Survival curves were estimated using the Kaplan–Meier method, and significant differences in survival were assessed using the log-rank test. Univariate and multivariate analyses with a Cox proportional hazards model were performed to determine the significant factors associated with OS. Multivariate analysis was performed for parameters with *p* <  0.05 in the univariate analysis. All statistical analyses were performed using StatFlex version 6 (Artech Co., Ltd., Osaka, Japan) and GraphPad Prism 8.0.2 (GraphPad Software, CA, USA), and *p* < 0.05 was considered statistically significant.

## Results

### Baseline patient characteristics

The clinicopathological characteristics of the 141 patients enrolled in this study are shown in Table 1. The mean age of patients (± standard deviation) was 52.3 ± 10.1 years, and all the patients were female. Seventy-nine (56.0%) patients were premenopausal, whereas 62 (44.0%) patients were postmenopausal. With regard to the histological type of breast cancer, 127 (90.1%) patients had invasive ductal carcinoma, and 14 (9.9%) patients had special types. As for HG, 38 (27.0%), 69 (48.9%), and 29 (20.6%) patients had breast cancer with HG 1, 2, and 3, respectively. Regarding the subtype of breast cancer, 75 (53.2%) cases were luminal, 30 (21.3%) cases were luminal HER2, 16 (11.3%) cases were HER2-enriched, and 20 (14.2%) cases were TN breast cancer. Axillary lymph nodes were involved in 122 (86.5%) patients. Regarding the clinical stage at diagnosis, 89 (63.1%) and 52 (36.9%) patients had stage II and III breast cancer, respectively. Fifty-seven (40.4%) patients were treated with EC/FEC followed by weekly PTX and/or trastuzumab, while 84 (59.6%) patients were with EC/FEC followed by triweekly DTX and/or trastuzumab. After NAC, mastectomy was performed in 107 (75.9%) patients, whereas partial resection of the breast was performed in 34 (24.1%) patients. pCR was obtained in 29 (20.6%) patients. The mean SMI before NAC (pre-SMI) was 46.5 ± 7.6, whereas that after NAC (post-SMI) was 46.3 ± 8.0. The mean BMI before NAC (pre-BMI) was 22.4 ± 3.7, and that after NAC (post-BMI) was 22.4 ± 3.7. The percent changes in SMI and BMI were − 27.7 to 19.6 and − 22.9 to 34.2, respectively. Regarding Alb and NLR, the mean values before NAC were 4.50 ± 0.30 and 2.53 ± 1.54, whereas those after NAC were 4.07 ± 0.36 and 3.03 ± 1.61, respectively. The changes in Alb and NLR were − 1.1 to 0.6 and − 5.20 to 6.00, respectively. The percent change in SMI was shown to have a slight inverse correlation with changes in both Alb and NLR values (Additional file 1**: Fig. S1a, b**). On the other hand, the percent change in SMI showed a tendency to have a positive correlation with the percent change in SMI (Additional file 1**: Fig. S1c**). The median follow-up period after surgery was 70 (range, 3–131) months, and 33 (23.4%) patients developed recurrence. Among these recurrences, locoregional relapses (chest wall, supraclavicular lymph nodes, or parasternal lymph nodes) were observed in 5 (3.5%) patients, while distant metastases (bone, lung, liver, or brain) occurred in 28 (19.9%) patients.

**Table 1:** Clinicopathological characteristics of patients

Variables	*n* = 141 (%)
Age (y.o. mean ± SD)	52.3 ± 10.1
Sex (Male/Female)	0/141
Menopausal status	
Premenopausal	79 (56.0)
Postmenopausal	62 (44.0)
Histological type	
Invasive ductal carcinoma	127 (90.1)
Special type	14 (9.9)
Histological grade	
1	38 (27.0)
2	69 (48.9)
3	29 (20.6)
Undetermined	5 (3.5)
Subtype	
Luminal	75 (53.2)
Luminal HER2	30 (21.3)
HER2-enriched	16 (11.3)
Triple negative	20 (14.2)
Lymph node metastasis	
Positive	122 (86.5)
Negative	19 (13.5)
Pre-NAC clinical stage	
II	89 (63.1)
III	52 (36.9)
Regimens of NAC	
EC/FEC → PTX and/or TRA	57 (40.4)
EC/FEC → DTX and/or TRA	84 (59.6)
Surgical procedures	
Mastectomy + Axillary dissection	107 (75.9)
Partial resection of breast + Axillary dissection	34 (24.1)
Pathological response to NAC	
Non-pCR	112 (79.4)
pCR	29 (20.6)
SMI (mean ± SD)	
Pre-NAC	46.5 ± 7.6
Post-NAC	46.3 ± 8.0
BMI (mean ± SD)	
Pre-NAC	22.4 ± 3.7
Post-NAC	22.4 ± 3.7
Percent change in SMI	−27.7 to 19.6
Percent change in BMI	−22.9 to 34.2
Alb (g/dl; mean ± SD)	
Pre-NAC	4.50 ± 0.30
Post-NAC	4.07 ± 0.36
NLR (mean ± SD)	
Pre-NAC	2.53 ± 1.54
Post-NAC	3.03 ± 1.61
Change in Alb	−1.1 to 0.6
Change in NLR	−5.20 to 6.00
Recurrence	
Total	33 (23.4)
Locoregional (Chest wall, ScLN, PsLN)	5 (3.5)
Distant (Bone, lung, liver, brain)	28 (19.9)

*NAC* Neoadjuvant chemotherapy, *HER2* Human epidermal growth factor receptor type 2, *EC* Epirubicin and cyclophosphamide, *FEC* Fluorouracil, epirubicin, and cyclophosphamide, *PTX* Paclitaxel, *DTX* Docetaxel, *TRA* Trastuzumab, *pCR* Pathological complete response, *SMI* Skeletal muscle index, *BMI* Body mass index, *Alb* Serum albumin level, *NLR* Neutrophil-to-lymphocyte ratio, *ScLN* Supraclavicular lymph node, *PsLN* Parasternal lymph node

### Association between skeletal muscle index (SMI) or body mass index (BMI) and recurrence

To analyze the influence of skeletal muscle mass and body weight on disease recurrence, we divided patients into two groups according to the presence of recurrence and compared pre- and post-SMI and BMI. There were no significant differences in either pre-SMI or pre-BMI between patients with recurrence and those without recurrence (*p* = 0.91 for pre-SMI, *p* = 0.70 for pre-BMI) (Additional file 2**: Fig. S2a, b**). In contrast, after NAC, patients who developed recurrence exhibited significantly lower post-SMI (*p* = 0.010), whereas post-BMI did not show a difference between the two groups (*p* = 0.88) (Additional file 2**: Fig. S2c, d**).

### Association between changes in SMI during neoadjuvant chemotherapy (NAC) and patient outcomes

**Fig. 1:**
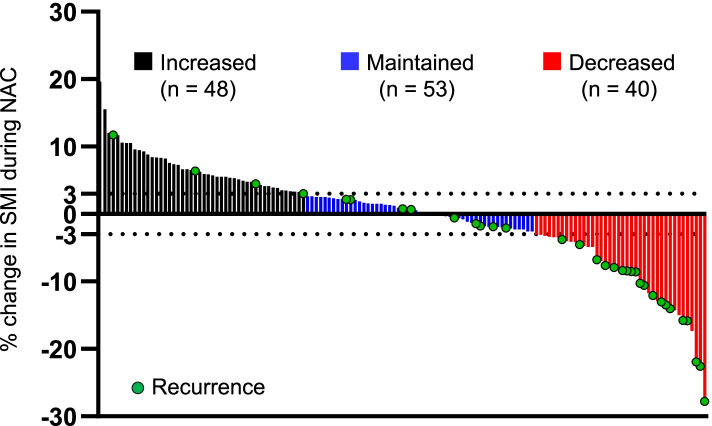
Percent Change in SMI during NAC of individual patient. Increased was defined as more than 3% increase, maintained as within − 3 to 3% change, and decreased as more than 3% decrease, respectively. Circle indicates the patient who developed recurrence. SMI: Skeletal muscle index, NAC: Neoadjuvant chemotherapy

As we found a significant difference in post-SMI between patients with and without recurrence after NAC, we focused on the changes in SMI during NAC in individual patients and their correlation with DFS and OS. To address this, we divided the patients into three groups (increased: ≥ 3% increase, maintained: − 3% <, < 3% change, decreased: − 3% ≥ decrease in SMI during NAC) according to the percent change in SMI. As shown in Fig. 1, 48 (34.0%), 53 (37.6%), and 40 (28.4%) patients exhibited increased, maintained, and decreased SMI, respectively. The clinicopathological characteristics of the decreased, maintained, and increased SMI groups are shown in Table 2 and Table 3. Menopausal status, histological type, HG, subtype, lymph node metastasis, clinical stage, NAC regimen, surgical procedure were not significantly different among the three groups, whereas the increased SMI group had a significantly lower mean age (*p* = 0.03) and higher pCR rate (*p* = 0.03) than the maintained and decreased SMI groups. Recurrence was significantly more prevalent in the decreased SMI group than in the maintained and decreased SMI groups (*p* = 0.04). Distant metastases were frequently developed in the decreased SMI group (45.0%) compared to the maintained (13.2%) and decreased (6.3%) SMI groups although there was no significant difference (*p* = 0.32). As shown in Table 3, pre-Alb, post-Alb, and change in Alb during NAC were not significantly different among the three groups. Although there was no statistically significant difference, pre-NLR and post-NLR values were the highest in the decreased SMI group (pre: 2.77 ± 2.13, post: 3.40 ± 1.79), and gradually decreased toward the maintained (pre: 2.49 ± 1.38, post: 3.02 ± 1.59) and increased (pre: 2.38 ± 1.04, post: 2.72 ± 1.44) SMI groups. Furthermore, the change in NLR was the highest in the decreased SMI group (0.62 ± 1.99) and showed a decreasing trend toward the maintained (0.54 ± 1.74) and increased (0.20 ± 1.31) SMI groups in this order. Regarding BMI, although pre-BMI and post-BMI were not significantly different between the three groups, percent change in BMI was highest in the increased SMI group (0.91 ± 5.74) and gradually decreased toward the maintained (− 0.22 ± 3.96) and decreased (− 1.11 ± 8.26) SMI groups.

**Table 2:** Comparison of clinicopathological features among the decreased, maintained, and increased SMI groups

	SMI			
	Decreased	Maintained	Increased	
Variables	*n* = 40 (%)	*n* = 53 (%)	*n* = 48 (%)	*p* value
Age (y.o. mean ± SD)	54.3 ± 11.3	53.7 ± 9.5	49.1 ± 9.0	0.03
Sex (Male/Female)	0/40	0/53	0/48	
Menopausal status				
Premenopausal	21 (52.5)	25 (47.2)	33 (68.8)	0.11
Postmenopausal	19 (47.5)	28 (52.8)	15 (31.2)	
Histological type				
Invasive ductal carcinoma	35 (87.5)	46 (86.8)	46 (95.8)	0.17
Special type	5 (12.5)	7 (13.2)	2 (4.2)	
Histological grade				
1	17 (42.5)	10 (18.9)	11 (22.9)	0.06
2	15 (37.5)	26 (49.1)	28 (58.3)	
3	8 (20.0)	15 (28.2)	6 (12.5)	
Undetermined	0 (0.0)	2 (3.8)	3 (6.3)	
Subtype				
Luminal	26 (65.0)	27 (50.9)	22 (45.8)	0.19
Luminal HER2	7 (17.5)	9 (16.9)	14 (29.2)	
HER2-enriched	5 (12.5)	5 (9.5)	6 (12.5)	
Triple negative	2 (5.0)	12 (22.7)	6 (12.5)	
Lymph node metastasis				
Positive	39 (97.5)	42 (79.2)	41 (85.4)	0.12
Negative	1 (2.5)	11 (20.8)	7 (14.6)	
Pre-NAC clinical stage				
II	23 (57.5)	39 (73.6)	27 (56.2)	0.81
III	17 (42.5)	14 (26.4)	21 (43.8)	
Regimens of NAC				
EC/FEC → PTX and/or TRA	16 (40.0)	20 (37.7)	21 (43.8)	0.70
EC/FEC → DTX and/or TRA	24 (60.0)	33 (62.3)	27 (56.2)	
Surgical procedures				
Bt + Ax	33 (82.5)	39 (73.6)	35 (72.9)	0.31
Bp + AX	7 (17.5)	14 (26.4)	13 (27.1)	
Pathological response to NAC				
Non-pCR	35 (87.5)	44 (83.0)	33 (68.8)	0.03
pCR	5 (12.5)	9 (17.0)	15 (31.2)	
Recurrence				
Total	20 (50.0)	9 (17.0)	4 (8.3)	0.04
Locoregional	2 (5.0)	2 (3.8)	1 (2.1)	0.32
Distant	18 (45.0)	7 (13.2)	3 (6.3)	

*SMI* Skeletal muscle index, *NAC* Neoadjuvant chemotherapy, *HER2* Human epidermal growth factor receptor type 2, *EC* Epirubicin and cyclophosphamide, *FEC* Fluorouracil, epirubicin, and cyclophosphamide, *PTX* Paclitaxel, *DTX* Docetaxel, *TRA* Trastuzumab, *Bt* Mastectomy, *Bp* Partial resection of breast, *Ax* Axillary dissection, *pCR* Pathological complete response

**Table 3:** Comparison of Alb, NLR, and BMI, and their change during NAC among the decreased, maintained, and increased SMI groups

	SMI			
	Decreased	Maintained	Increased	
Variables	*n =* 40	*n =* 53	*n =* 48	*p* value
Pre-Alb (g/dl)	4.59 ± 0.29	4.51 ± 0.28	4.42 ± 0.31	0.65
Post-Alb (g/dl)	4.20 ± 0.34	4.01 ± 0.31	4.03 ± 0.43	0.52
Change in Alb (mean ± SD)	−0.31 ± 0.38	−0.44 ± 0.34	−0.33 ± 0.36	0.18
Pre-NLR	2.77 ± 2.13	2.49 ± 1.38	2.38 ± 1.04	0.48
Post-NLR	3.40 ± 1.79	3.02 ± 1.59	2.72 ± 1.44	0.14
Change in NLR (mean ± SD)	0.62 ± 1.99	0.54 ± 1.74	0.20 ± 1.31	0.71
Pre-BMI	22.2 ± 3.51	22.0 ± 2.98	22.7 ± 4.10	0.64
Post-BMI	22.0 ± 3.51	22.0 ± 3.10	22.9 ± 4.45	0.64
Percent change in BMI (mean ± SD)	−1.11 ± 8.26	−0.22 ± 3.96	0.91 ± 5.74	0.28

*Alb* Serum albumin level, *NLR* Neutrophil-to-lymphocyte ratio, *BMI* Body mass index, *NAC* Neoadjuvant chemotherapy, *SMI* Skeletal muscle index

**Fig. 2:**
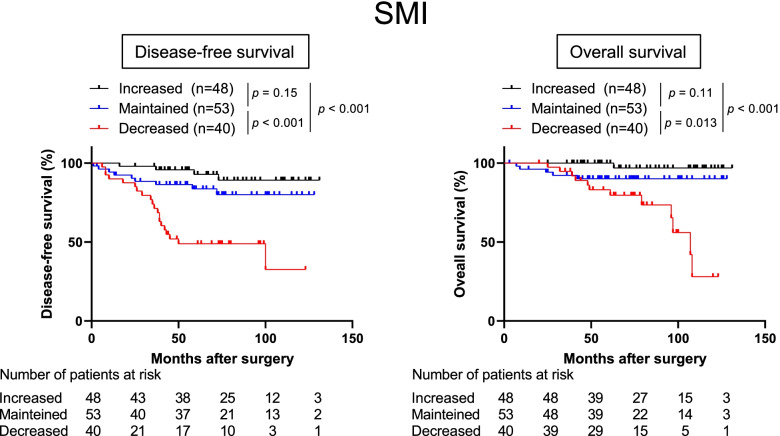
Kaplan–Meier curves for DFS (left) and OS (right) according to changes in SMI (increased, maintained, and decreased). DFS, HR 8.29; 95%CI 3.63–18.9; *p* < 0.001 for decreased vs. increased, HR 3.49; 95%CI 1.65–7.36; *p* < 0.001 decreased vs. maintained, HR 2.30; 95%CI 0.77–6.84; *p* = 0.15 maintained vs. increased. OS, HR 19.8; 95%CI 6.43–60.6; *p* < 0.001 for decreased vs. increased, HR 3.41; 95%CI 1.29–8.95; *p* = 0.013 decreased vs. maintained, HR 4.98; 95%CI 0.99–24.5; *p* = 0.11, maintained vs. increased). DFS: Disease-free survival, OS: Overall survival, SMI: Skeletal muscle index, NAC: Neoadjuvant chemotherapy, HR: Hazard ratio, CI: Confidence interval

The decreased SMI group had significantly poorer DFS than the maintained and increased groups (hazard ratio [HR] 8.29, 95% confidence interval [CI] 3.63–18.9, *p* < 0.001 for the decreased vs. increased SMI group; HR 3.49, 95% CI 1.65–7.36, *p* < 0.001 for the decreased vs. maintained SMI group) (Fig. 2). In addition, a trend for poorer DFS was found in the maintained SMI group than in the increased SMI group, although the difference was not statistically significant (HR 2.30, 95% CI 0.77–6.84, *p* = 0.15) (Fig. 2). In line with the DFS, the OS was significantly shorter in the decreased SMI group than in the maintained and increased SMI groups (HR 19.8, 95% CI 6.43–60.6, *p* < 0.001 for the decreased vs. increased SMI group; HR 3.41, 95% CI 1.29–8.95, *p* = 0.013 for the decreased vs. maintained SMI group) (Fig. 2).

### Association between SMI change during NAC and patient outcomes on various clinical factors

To investigate whether the effect of SMI change on prognosis is dependent on various clinical factors, including menopausal status (premenopausal vs. postmenopausal), pretreatment clinical stage (stage II vs. stage III), and subtype of breast cancer, we divided the patients according to these factors and compared the DFS among the decreased, maintained, and increased SMI groups with each factor. We found that the decreased SMI group showed poorer DFS than the maintained and increased SMI groups regardless of menopausal status and clinical stage (Additional file 3**: Fig. S3;** Additional file 4**: Table S1**). With regard to subtype, the decreased SMI group showed significantly worse DFS than the increased SMI group in patients with luminal (*p* < 0.001), HER2-enriched (*p* = 0.020), and TN (*p* = 0.039) breast cancer. Of the 30 patients with luminal HER2 breast cancer, only two developed recurrence. Possibly due to the lack of patients who had recurrence, we did not find statistical significance in DFS between the decreased and increased groups in this subtype. However, one patient who exhibited disease recurrence was in the decreased group (Additional file 5**: Fig. S4,** Additional file 4**: Table S1**).

Next, we investigated whether the kind of taxane regimen (PTX or DTX) would affect the prognosis or impact of SMI change, and found that there was no significant difference in DFS between the patients treated with PTX and those treated with DTX (HR 0.95, 95% CI 0.47–1.91, *p* = 0.89) (Additional file 6**: Fig. S5**). Furthermore, irrespective of taxane regimen, the decreased SMI group exhibited poorer DFS than the maintained and increased SMI groups (Additional file 7**: Fig. S6,** Additional file 4**: Table S1**).

### Univariate and multivariate analyses

To confirm the significance of changes in SMI on DFS, univariate and multivariate analyses were performed. Univariate analysis revealed that a decrease in SMI was significantly associated with poorer DFS (HR 3.46, 95% CI 1.56–7.65, *p* < 0.01). The other factors that correlated with poorer DFS were postmenopausal (HR 2.15, 95% CI 1.07–4.34, *p* = 0.03), luminal HER2 (HR 0.20, 95% CI 0.04–0.88, *p* = 0.03), pre-NAC clinical stage III (HR 2.18, 95% CI 1.10–4.32, *p* = 0.02), and pCR (HR 0.22, 95% CI 0.05–0.95, *p* = 0.04). On multivariate analysis using the Cox hazards model, SMI decrease was an independent predictive factor for poorer DFS (HR 3.68, 95% CI 1.54–9.01, *p* < 0.01) (Table 4).

**Table 4:** Univariate and multivariate Cox proportional hazards regression analyses of the clinicopathological parameters

	Univariate			Multivariate		
	HR	95% CI	*p* value	HR	95% CI	*p* value
Age						
< 50	1.00					
≥ 50	2.03	0.96–4.28	0.06			
Menopausal status						
Premenopausal	1.00			1.00		
Postmenopausal	2.15	1.07–4.34	0.03	2.27	1.07–4.78	0.03
Histological type						
Invasive ductal carcinoma	1.00					
Special type	0.61	0.23–1.58	0.30			
Subtype						
Luminal	1.00			1.00		
Luminal HER2	0.20	0.04–0.88	0.03	0.19	0.04–0.82	0.03
HER2 enriched	0.92	0.31–2.70	0.89	1.01	0.32–3.15	0.97
TNBC	1.20	0.48–2.98	0.68	2.20	0.80–6.06	0.12
Pre-NAC clinical stage						
Stage II	1.00			1.00		
Stage III	2.18	1.10–4.32	0.02	2.43	1.18–5.00	0.02
Pathological response to NAC						
Non-pCR	1.00					
pCR	0.22	0.05–0.95	0.04	0.34	0.07–1.54	0.16
SMI						
Maintained	1.00			1.00		
Increased	0.43	0.13–1.40	0.16	0.49	0.14–1.72	0.20
Decreased	3.46	1.56–7.65	< 0.01	3.68	1.52–8.96	< 0.01
BMI						
Maintained	1.00					
Increased	1.61	0.67–3.86	0.28			
Decreased	1.26	0.54–2.90	0.58			
Alb						
Increased	1.00					
Decreased	1.14	0.49–2.63	0.75			
NLR						
Increased	1.00					
Decreased	1.10	0.51–2.21	0.77			

*NAC* Neoadjuvant chemotherapy, *HER2* Human epidermal growth factor receptor type 2, *pCR* Pathological complete response, *SMI* Skeletal muscle mass index, *BMI* Body mass index, *Alb*: Serum albumin level, *NLR* Neutrophil-to-lymphocyte ratio

## Discussion

The present study demonstrates that skeletal muscle loss during NAC is significantly associated with poor prognosis and is an independent predictor of DFS in patients with breast cancer. In addition, the results of this study suggest that SMI decrease might be a prognostic marker of patient outcomes irrespective of menopausal status, clinical stage, and subtype of breast cancer. To the best of our knowledge, this is the first study to demonstrate that loss of skeletal muscle is a prognostic factor in patients with breast cancer who underwent NAC.

Accumulating evidence suggests that patients with cancer who underwent palliative chemotherapy were likely to lose skeletal muscle mass in various solid malignancies, including gastric, colorectal, and lung cancers [17–19, 22, 25, 26]. Furthermore, recent studies in patients with esophageal cancer have shown that not only palliative chemotherapy but also NAC comprising fluorouracil, cisplatin, and adriamycin or fluorouracil, cisplatin, and DTX could decrease SMI [25, 27, 28]. However, as for breast cancer, there have been no studies demonstrating the loss of SMI during NAC. In this regard, the results of this study showed that 27.4% of all patients lost more than 3% of SMI during NAC, which provides a novel insight that anthracycline and taxane-based NAC for breast cancer can induce skeletal muscle loss.

Skeletal muscle atrophy has been known to be caused by both reduction of protein synthesis and protein degradation in various diseases and body conditions [29]. Three possible mechanisms underlying muscle loss during chemotherapy have been considered: (1) decreased food intake due to gastrointestinal adverse effects, (2) reduced physical activity secondary to general fatigue, and (3) direct effect of chemotherapy on muscles [26]. Furthermore, as another mechanism, the indirect effect of chemotherapy on muscle alteration via immune cells was suggested [30]. Wang et al. showed that T cells could attenuate muscle mass loss during cancer progression [31]. In contrast, circulating neutrophils in patients with cancer have been shown to induce skeletal muscle degeneration [30, 32]. Although the severity varies in each patient, chemotherapy generally causes neutropenia and lymphopenia [33] and thus can indirectly affect muscle alteration. In this regard, a decrease in NLR may prevent muscle loss during NAC. This notion is consistent with our results, showing that percent change in SMI had a moderate negative correlation with the change in NLR value, indicating that patients with decreased SMI tended to have increased NLR during NAC. On the other hand, the results of the present study demonstrate that the percent change in SMI did not have a positive correlation with change in serum Alb. This suggests that NAC-induced malnutritional status might not be the main cause of muscle loss. Therefore, although further investigations are needed, the present study indicates that the muscle loss during NAC for patients with breast cancer may be due, at least in part, to the indirect effects of chemotherapy on muscles via immune cells.

Although emerging evidence suggests that pretreatment SMI is a prognostic marker for patients with breast cancer [8, 10, 13, 14, 34–37], the clinical significance of changes in SMI during treatment in an individual patient remains unknown. In this regard, our results provide a novel finding that a decrease in SMI during NAC might have a negative effect on prognosis in patients with breast cancer. Similar to this study, we previously demonstrated that a decrease in PNI during NAC for patients with breast cancer was associated with worse prognosis, but not pretreatment PNI [3]. Furthermore, other studies have shown that changes in NLR during NAC correlated with the efficacy of NAC and patient survival [38–40]. These reports, together with the results of the present study, highlight the importance of monitoring the dynamic changes in systemic nutritional conditions and body composition throughout the treatment course.

The results of our study indicate that SMI loss would have high availability as a prognostic factor in patients with breast cancer who underwent NAC. First, poorer outcomes in patients with decreased SMI were observed irrespective of menopausal status and pretreatment clinical stage. Thus, the usefulness of SMI loss as a prognostic factor was indicated regardless of the hormonal conditions of patients, patient age, and tumor burden at the beginning of NAC. Second, poorer outcomes in patients with decreased SMI were observed regardless of the subtype. Generally, the skeletal muscle volume of patients with breast cancer may be independent of tumor characteristics determined by ER and HER2 expression, especially in the early stages [4]. The results of the present study are consistent with this notion and indicate that SMI change can predict patient outcomes irrespective of tumor biology itself. Altogether, our results suggest that SMI loss during NAC can be a prognostic marker in any age, stage, or subtype. However, majority of patients enrolled in this study had luminal type breast cancer, and the number of HER2-positive or TN breast cancer was limited. Luminal type breast cancer is known to show limited response to chemotherapy; hence, it is difficult to accomplish pCR, whereas HER2-positive or TN breast cancer has high sensitivity to chemotherapy as a subset of these subtypes can be pathologically eliminated (pCR) by NAC [2]. Thus, the impact of SMI change on response to chemotherapy varies according to the subtype. Regarding the influence of SMI change on DFS of patients who underwent NAC, although a similar trend was observed among luminal, HER2-enriched, and TN breast cancer in this study, further large-scale studies are required to determine the importance of SMI change in each subtype of breast cancer.

Our results highlight the importance of SMI maintenance during NAC. To date, phase III studies (ROMANA1 and ROMANA 2) have demonstrated that pharmacotherapies with anamorelin (a ghrelin receptor agonist) significantly increased lean body mass in patients with non-small cell lung cancer who underwent chemotherapy [41]. However, multimodal approaches are required to dissolve multifactorial symptoms, such as SMI loss [42]. Indeed, a phase III trial (NCT02330926) is currently ongoing to test whether a multimodal intervention, including exercise, nutritional support, and anti-inflammatory medication, plus standard care can improve the treatment outcome of patients with cancer, the results of which are awaited.

This study has some limitations. First, it was a retrospective analysis with a relatively small study population in a single institution from one Asian country. Second, the NAC regimens were not uniform among patients because the study period spanned several years when the treatment regimens were changed. Furthermore, majority of patients enrolled in this study had luminal subtype breast cancer and were perimenopausal. Thus, estrogen-related effect can have some impact on the results of this study. Although our results indicate that a decrease in SMI is associated with poor outcomes irrespective of menopausal status and subtype, we could not determine the impact of estrogen-driven factors from our results. Chemotherapy-induced menopause also should be taken into consideration. To overcome these limitations and validate our results, further investigations are required. In particular, a prospective multicountry study is warranted to diversify our findings to more heterogenous populations with different races/ethnicities and environmental backgrounds.

## Conclusions

The findings of the present study indicate that skeletal muscle loss during NAC can be a prognostic marker in patients with breast cancer. If this observation is confirmed in larger prospective studies from other countries, it could provide the basis of the importance to monitor and maintain skeletal muscle mass during NAC.

## Supplementary Information

**Additional file 1.**

**Additional file 2.**

**Additional file 3.**

**Additional file 4.**

**Additional file 5.**

**Additional file 6.**

**Additional file 7.**

## Data Availability

The data supporting the findings of this work are available from the corresponding author upon reasonable request.

## Abbreviations

Alb: Serum albumin level
BMI: Body mass index
DTX: Docetaxel
EC: Epirubicin and cyclophosphamide
ER: Estrogen receptor
FNA: Fine-needle aspiration cytology
FEC: Fluorouracil epirubicin and cyclophosphamide
HER2: Human epidermal growth factor receptor type 2
HG: Histological grade
NAC: Neoadjuvant chemotherapy
NLR: Neutrophil-to-lymphocyte ratio
pCR: Pathological complete response
PgR: Progesterone receptor
PNI: Prognostic nutritional index
PTX: Paclitaxel
ROC: Receiver operating characteristics
SMA: Skeletal muscle area
SMI: Skeletal muscle index
TN: Triple negative
