# Supplementary material for: Skeletal muscle loss during neoadjuvant chemotherapy predicts poor prognosis in patients with breast cancer
Source: BMC Cancer. 2022 Mar 26;22:327. doi: 10.1186/s12885-022-09443-1 (PMC8962250; doi:10.1186/s12885-022-09443-1)
Supplement: Supplementary file 1 — Additional file 1. [file 12885_2022_9443_MOESM1_ESM.pdf]

Fig.S1

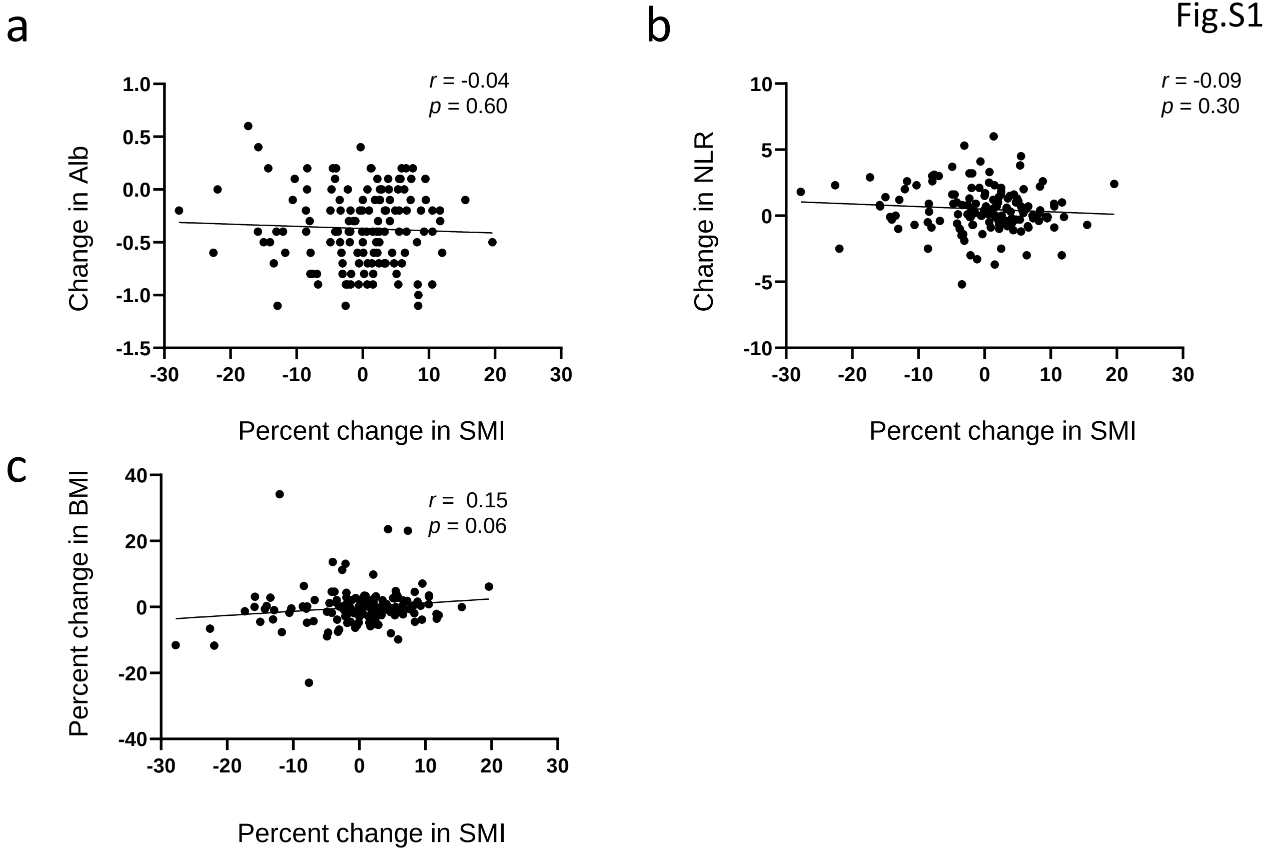

Fig. S1. Scatter plot of change in Alb value (a), that in NLR value (b), and percent change in BMI (c) against percent change in SMI. Correlation is shown using Pearson correlation (r).
Alb: Serum albumin level (g/dl), NLR: Neutrophil-to-lymphocyte ratio, SMI: Skeletal muscle index, BMI: Body mass index
