# Supplementary material for: Skeletal muscle loss during neoadjuvant chemotherapy predicts poor prognosis in patients with breast cancer
Source: BMC Cancer. 2022 Mar 26;22:327. doi: 10.1186/s12885-022-09443-1 (PMC8962250; doi:10.1186/s12885-022-09443-1)
Supplement: Supplementary file 2 — Additional file 2. [file 12885_2022_9443_MOESM2_ESM.pdf]

Fig.S2

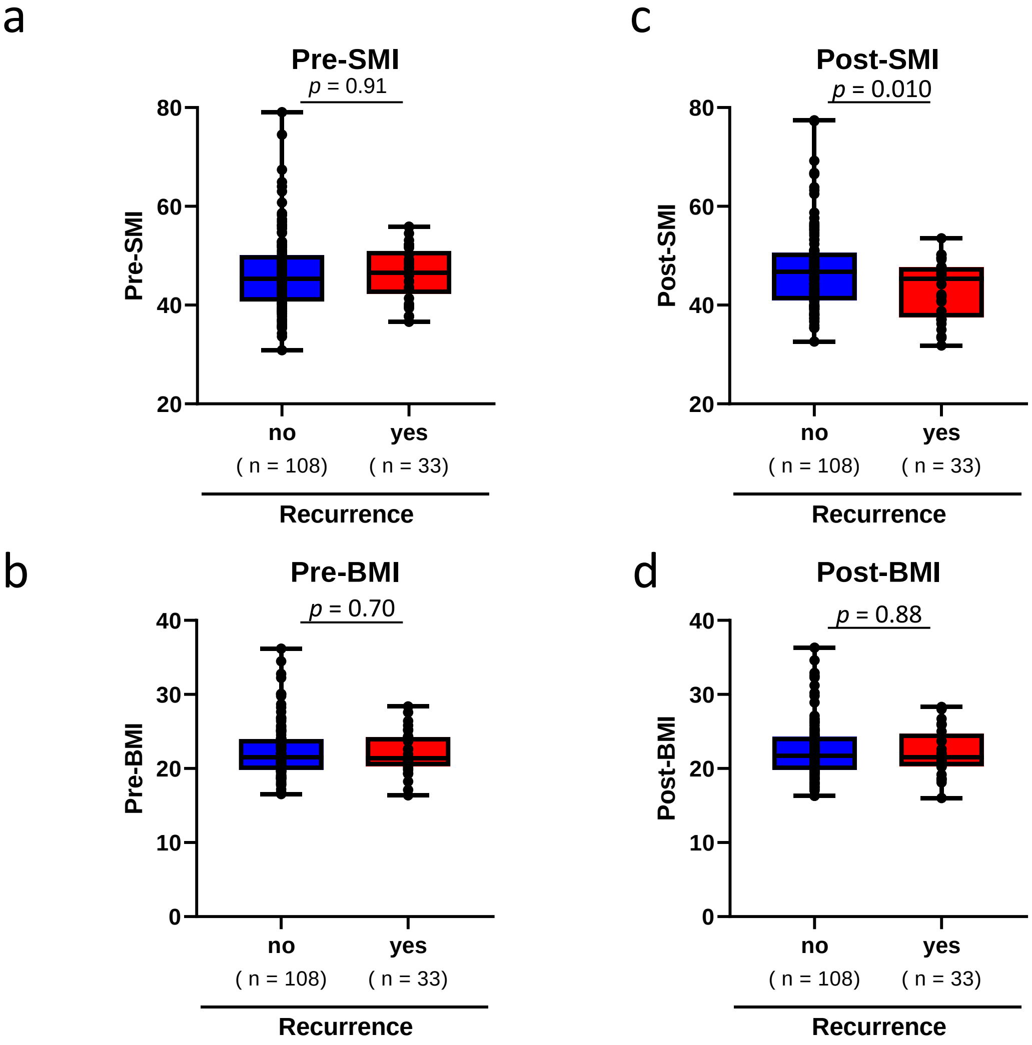

Fig. S2. Box-and-whisker plot for SMI (a) and BMI (b) before NAC (a; Pre-SMI, b; Pre-BMI) and after NAC (a; Post-SMI, b; Post-BMI) in patients with and without recurrence.
NAC: Neoadjuvant chemotherapy, SMI: Skeletal muscle index, BMI: Body mass index
