# Supplementary material for: Skeletal muscle loss during neoadjuvant chemotherapy predicts poor prognosis in patients with breast cancer
Source: BMC Cancer. 2022 Mar 26;22:327. doi: 10.1186/s12885-022-09443-1 (PMC8962250; doi:10.1186/s12885-022-09443-1)
Supplement: Supplementary file 3 — Additional file 3. [file 12885_2022_9443_MOESM3_ESM.pdf]

Fig.S3

a

Premenopausal

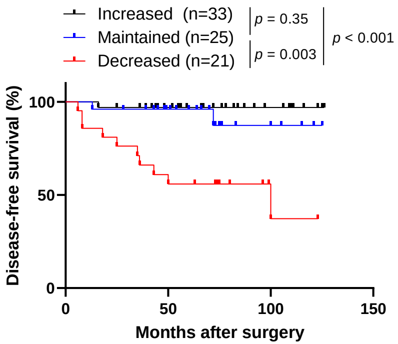

Number of patients at risk

Increased	33	32	26	19	9	2
Mainteined	25	24	18	9	6	1
Decreased	21	17	12	8	3	1

Postmenopausal

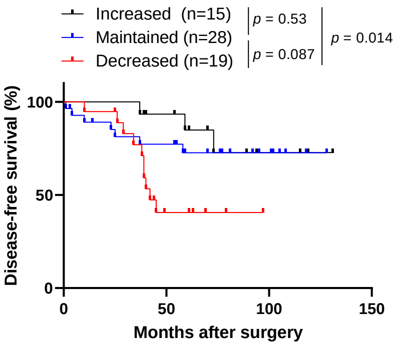

Number of patients at risk

Increased	15	15	13	9	4	2
Mainteined	28	22	20	13	8	2
Decreased	19	18	6	3	1	

b

Stage II

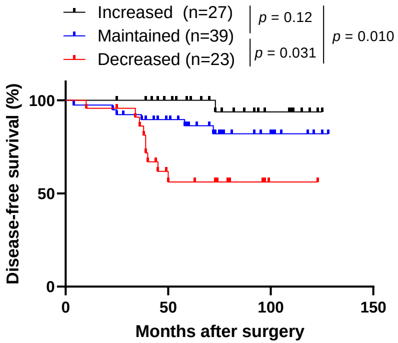

Number of patients at risk

Increased	27	27	23	16	8	1
Mainteined	39	37	30	18	11	2
Decreased	23	22	11	8	2	1

Stage III

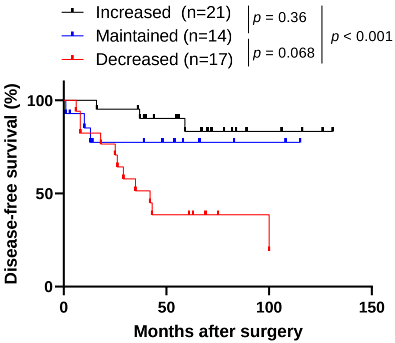

Number of patients at risk

Increased	21	21	16	12	5	3
Mainteined	14	10	8	4	3	
Decreased	17	13	7	3	2	

Fig. S3. Kaplan–Meier curves for DFS according to changes in SMI (increased, maintained, and decreased) by menopausal status (a) and clinical stage (b).
DFS: Disease-free survival, SMI: Skeletal muscle index
