# Supplementary material for: Skeletal muscle loss during neoadjuvant chemotherapy predicts poor prognosis in patients with breast cancer
Source: BMC Cancer. 2022 Mar 26;22:327. doi: 10.1186/s12885-022-09443-1 (PMC8962250; doi:10.1186/s12885-022-09443-1)
Supplement: Supplementary file 4 — Additional file 4. [file 12885_2022_9443_MOESM4_ESM.pdf]

Table S1: Hazard ratio and 95% confidence interval in comparison of disease-free survival among to increased, maintained, and decreased skeletal muscle index groups according to menopausal status, clinical stage, subtype, and taxane regimen.

Variables		HR	95% CI	*p* value
Premenopausal	Increased	1.00		
	Maintained	2.93	0.29 – 29.2	0.35
	Decreased	19.3	5.55 – 67.1	< 0.001
Postmenopausal	Increased	1.00		
	Maintained	1.52	0.43 – 5.41	0.53
	Decreased	4.28	1.43 – 12.8	0.01
Stage II	Increased	1.00		
	Maintained	4.58	1.01 – 20.1	0.12
	Decreased	13.8	3.89 – 49.3	0.01
Stage III	Increased	1.00		
	Maintained	2.05	0.37 – 11.3	0.36
	Decreased	6.40	2.15 – 19.1	< 0.001
Luminal	Increased	1.00		
	Maintained	1.84	0.37 – 9.133	0.47
	Decreased	9.38	3.60 – 24.4	< 0.001
Luminal HER2	Increased	1.00		
	Maintained	NA	NA	0.42
	Decreased	2.42	0.11 – 51.2	0.51
HER2-enriched	Increased	1.00		
	Maintained	1.83	0.10 – 33.4	0.65
	Decreased	4.10	0.36 – 45.7	0.02
Triple negative	Increased	1.00		
	Maintained	NA	NA	0.10
	Decreased	NA	NA	0.04
Paclitaxel	Increased	1.00		
	Maintained	3.03	0.68 – 13.4	0.16
	Decreased	6.57	1.66 – 25.9	< 0.01
Docetaxel	Increased	1.00		
	Maintained	1.75	0.35 – 8.73	0.50
	Decreased	9.47	3.38 – 26.5	< 0.001

HER2: Human epidermal growth factor receptor type 2, NA: Not applicable, HR: Hazard ratio, CI: Confidence interval
