# Supplementary material for: Skeletal muscle loss during neoadjuvant chemotherapy predicts poor prognosis in patients with breast cancer
Source: BMC Cancer. 2022 Mar 26;22:327. doi: 10.1186/s12885-022-09443-1 (PMC8962250; doi:10.1186/s12885-022-09443-1)
Supplement: Supplementary file 5 — Additional file 5. [file 12885_2022_9443_MOESM5_ESM.pdf]

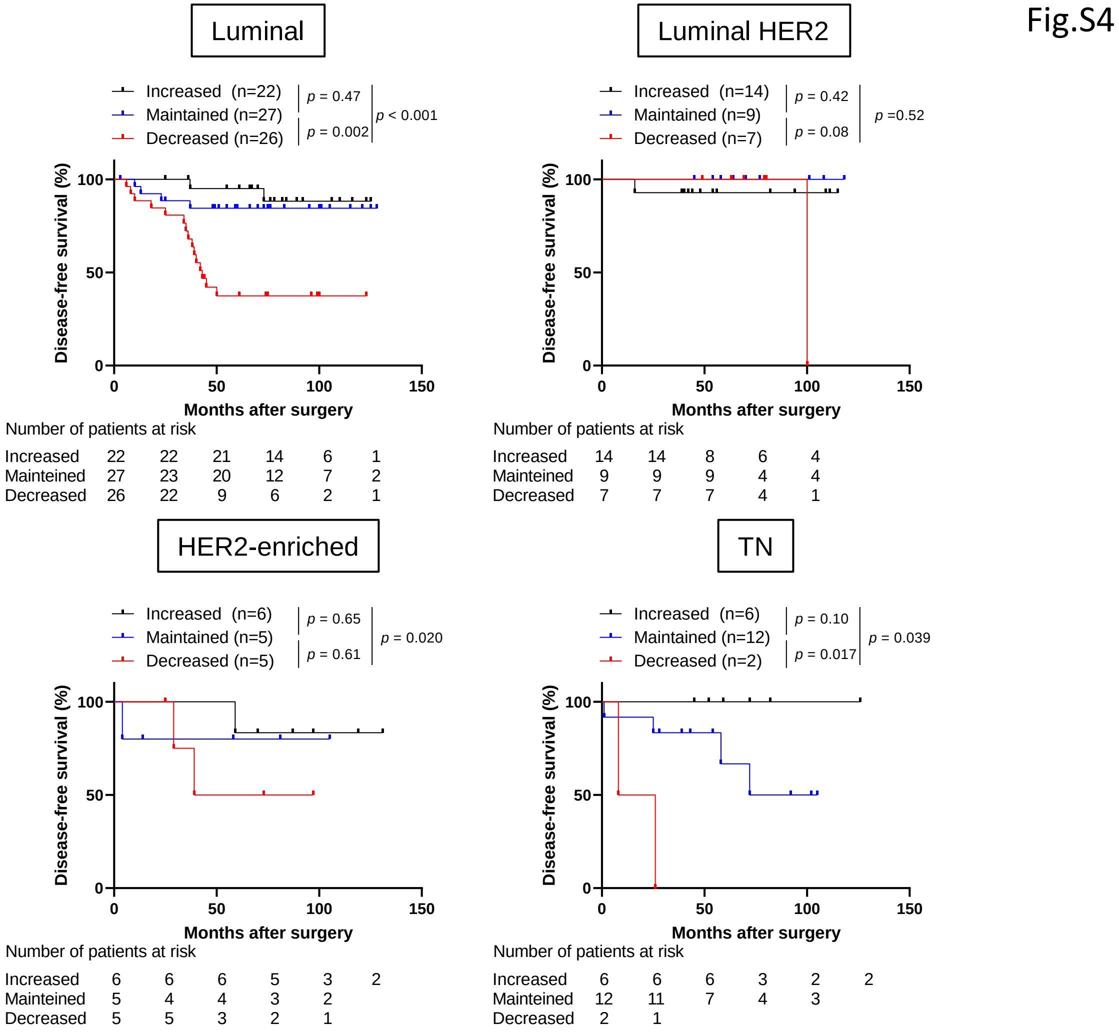

Fig. S4. Kaplan–Meier curves for DFS according to changes in SMI (increased, maintained, and decreased) by subtype of breast cancer.
DFS: Disease-free survival, SMI: Skeletal muscle index, HER2: Human epidermal growth factor receptor type 2, TN: Triple negative
