# Supplementary material for: Skeletal muscle loss during neoadjuvant chemotherapy predicts poor prognosis in patients with breast cancer
Source: BMC Cancer. 2022 Mar 26;22:327. doi: 10.1186/s12885-022-09443-1 (PMC8962250; doi:10.1186/s12885-022-09443-1)
Supplement: Supplementary file 6 — Additional file 6. [file 12885_2022_9443_MOESM6_ESM.pdf]

Fig.S5

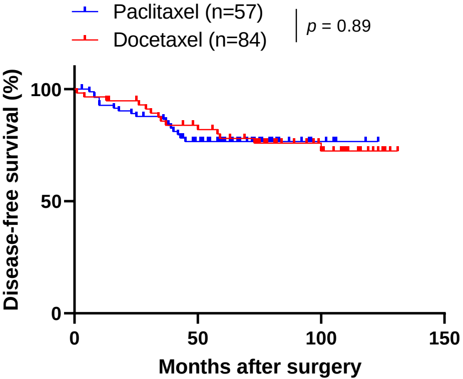

Number of patients at risk

Paclitaxel	57	53	44	33	22	5
Docetaxel	84	73	47	21	7	1

Fig. S5. Kaplan–Meier curves for DFS of patients treated with paclitaxel and docetaxel. DFS: Disease-free survival
