# Supplementary material for: Skeletal muscle loss during neoadjuvant chemotherapy predicts poor prognosis in patients with breast cancer
Source: BMC Cancer. 2022 Mar 26;22:327. doi: 10.1186/s12885-022-09443-1 (PMC8962250; doi:10.1186/s12885-022-09443-1)
Supplement: Supplementary file 7 — Additional file 7. [file 12885_2022_9443_MOESM7_ESM.pdf]

Fig.S6

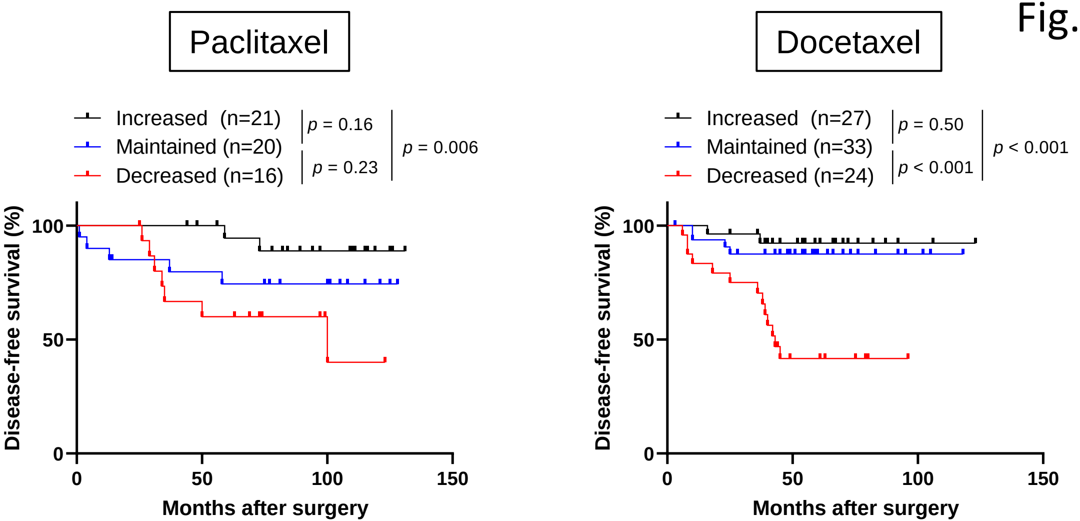

Number of patients at risk

Increased	21	21	20	17	10	3
Mainteined	20	20	16	16	11	2
Decreased	16	15	10	6	4	

Number of patients at risk

Increased	27	26	19	9	3	1
Mainteined	33	29	21	9	4	
Decreased	24	19	8	5	2	

Fig. S6. Kaplan–Meier curves for DFS according to changes in SMI (increased, maintained, and decreased) in patients treated with paclitaxel (left) and docetaxel (right).
DFS: Disease-free survival, SMI: Skeletal muscle index
